# Urgent need for writing education in schools of medicine and public health to address vaccine hesitancy

**DOI:** 10.5116/ijme.612d.ed97

**Published:** 2021-09-27

**Authors:** Tsuyoshi Okuhara, Hiroko Okada, Eiko Goto, Takahiro Kiuchi

**Affiliations:** 1 Department of Health Communication, School of Public Health, The University of Tokyo, Tokyo, Japan

**Keywords:** Writing education, schools of medicine, public health, vaccine hesitancy

## To the Editor

Vaccines have long been lauded as one of the most important public health achievements of the past century. In the past decade, however, a growing number of individuals have perceived vaccination as risky. Vaccine hesitancy, defined as “delay in acceptance or refusal of vaccines despite availability of vaccination service,” is a problem attracting growing attention and concern.^1^ As of 29 April 2021, approximately 150 million cases of COVID-19 infection and 3.14 million deaths owing to COVID-19 had been reported globally.^2^ Vaccination is an important tool to prevent further illness and death and to control the COVID-19 pandemic. The development of vaccines against COVID-19 has proceeded at an unprecedented pace. However, COVID-19 vaccine hesitancy is becoming a global problem.^3^^-^^5^ It is reported that about half of the population are hesitant or refuse to receive COVID-19 vaccines in countries such as the United States, the United Kingdom, Italy, France, Russia, Poland, Hong Kong, Jordan, and Kuwait.^3^^-^^5^

Communication can be an effective tool if used as part of a planned and integrated strategy to counteract vaccine hesitancy and promote optimal vaccine uptake.^6^ Communication involves both the message (i.e., what is said) and the delivery (i.e., how it is said).^7^ Previous studies have shown that written vaccination information by health professionals is inadequate both in the message and in the delivery. Studies show that anti-vaccination messages frequently use influential narratives from people who claim to have experienced adverse reactions as well as conspiracy theories; these types of messages are easy for lay audiences to understand.^8^^-^^11^ Pro-vaccination messages by health professions, however, frequently include probabilities and relative frequency statistics that are difficult for lay audiences to understand.^8^^,^^9^^,^^11^ Additionally, studies show that the readability level of vaccine information provided by health professionals is higher than an 8th-grade level,^12^^-^^15^ despite the fact that patient educational materials are recommended to be written at a 5th to 6th-grade level or lower.^16^ Studies using readability formulas have also shown that pro-vaccination messages were significantly more challenging to read than anti-vaccination messages.^17^^,^^18^ Furthermore, studies using the Patient Education Materials Assessment Tool (PEMAT) show that most vaccine information provided by health professionals does not meet the PEMAT threshold score for high understandability.^19^^,^^20^

To date, the most commonly studied intervention for vaccine hesitancy has used written educational information (e.g., brochures, pamphlets, and posters).^21^^,^^22^ Information from health care providers is one of the most influential factors in changing the opinions of vaccine-hesitant individuals.^23^ In such cases, written vaccine information follows a standardized approach to counseling and helps to ensure that barriers are addressed; furthermore, this approach plays an important role in individuals’ decision-making about vaccination.^20^ Understanding vaccine information requires a certain level of literacy and numeracy, making it challenging to convey vaccine information to lay individuals, especially if they have low health literacy.^24^ Thus, written vaccine information should be readable, understandable, and compelling.

In recent years, schools and programs in public health have focused on writing as a necessary skill, and faculties have recognized that students must be able to write clearly and effectively.^25^ In 2016 in the United States, the Council on Education for Public Health revised the Accreditation Criteria for Schools of Public Health and Public Health Programs and included, for the first time, criteria and requirements for written and oral communications at the levels of bachelor’s degree, master of public health degree, and doctor of public health degree.^25^ Schools of public health have begun programs to improve writing competency among their students. For example, the Harvard T.H. Chan School of Public Health,^25^ the University of Washington School of Public Health,^26^ and the Boston University School of Public Health^27^ have built extensive writing programs into their curriculum. In 2016, the authors also initiated a program to teach writing for public health in our series of health communication lectures at the School of Public Health at the University of Tokyo, which was the first program to teach writing for public health in Japan. However, to our knowledge, writing education for public health is only now beginning in some schools of public health in some countries.

Although writing for public health is one of the core competencies for health professionals, it receives little emphasis in education within schools of medicine and public health.^7^^,^^28^^-^^31^ This could be part of the reason why health professionals have been unable to apply the lessons learned from difficult experiences with communication regarding MMR and HPV vaccination as well as COVID-19 vaccination. COVID-19 vaccine hesitancy has exposed weaknesses in schools of medicine and public health that have not given sufficient attention to writing education aimed at improving vaccine-related communication and address vaccine hesitancy.

In medical education, a program should be developed that is focused on what, who, and how to teach writing to address vaccine hesitancy. Regarding what to teach, referring to studies of persuasion in social psychology, it is necessary to foster the ability to write messages that attract the interest of vaccine-hesitant individuals, convey information in a clear and memorable manner, and encourage vaccination.^32^ It will be helpful to teach basic frameworks, strategies, and theories to address vaccine hesitancy,^33^^,^^34^ persuasion techniques such as use of narratives (e.g., stories of patients with infectious diseases),^35^^,^^36^ and general heuristic rules such as social norms–if many others are doing it, it must be good (e.g., 4 out of 5 people are vaccinated).^37^^,^^38^ It will also be useful to introduce practical resources such as vaccination decision-aid tools that explain the risks and benefits of a vaccination decision in a personalized way.^39^ Regarding who should teach such a program, in addition to scholars in areas such as public health and social psychology, professionals in the field of advertising and marketing may be suitable lecturers on educating how to attract interest and use social media. A journalist may be a good lecturer on how to write concisely and clearly, as well as how to write a press release. Regarding how such a program should be taught, a writing-to-learn approach may be useful, one that helps students process and integrate new information into their understanding through short, low-stakes writing assignments.^40^^,^^41^ A writing-to-learn approach will encourage students to explore ideas regarding messages to encourage vaccination, synthesize the knowledge they have acquired in the program using their ideas, and discover the possibility of writing persuasive materials using innovative ideas. Much effort is needed in this arena. Providing writing education aimed at addressing vaccine hesitancy to medical students, postgraduate students, and health professionals is a crucial matter at present and in the future.

### Conflicts of Interest

The authors declare that they have no conflict of interest.
